# Supplementary material for: In vivo generation of human CD19‐CAR T cells results in B‐cell depletion and signs of cytokine release syndrome
Source: EMBO Mol Med. 2018 Sep 17;10(11):e9158. doi: 10.15252/emmm.201809158 (PMC6220327; doi:10.15252/emmm.201809158)
Supplement: Supplementary file 1 — Appendix [file EMMM-10-e9158-s001.docx]

**APPENDIX**

***In vivo* generation of human CD19-CAR T cells results in B cell depletion and signs of cytokine release syndrome**

Anett Pfeiffer^1,*^, Frederic B. Thalheimer^1,*^, Sylvia Hartmann^2^, Annika M. Frank^1^, Ruben R. Bender^1^, Simon Danisch^3^, Caroline Costa^4^, Winfried S. Wels^5,6,7^, Ute Modlich^8^, Renata Stripecke^3^, Els Verhoeyen^4,9^, Christian J. Buchholz^1,7,10,#^

^1^Molecular Biotechnology and Gene Therapy, Paul-Ehrlich-Institut, Langen, Germany.

^2^Dr. Senckenberg Institute of Pathology, Goethe-University Frankfurt, Frankfurt am Main, Germany

^3^Department of Hematology, Hemostasis, Oncology and Stem Cell Transplantation, Laboratory of Regenerative Immune Therapies Applied, Excellence Cluster REBIRTH and German Centre for Infection Research (DZIF), Partner site Hannover, Hannover, Germany.

^4^CIRI – International Center for Infectiology Research, Team EVIR, Inserm, U1111, Université Claude Bernard Lyon 1, CNRS, UMR5308, Ecole Normale Supérieure de Lyon, Univ Lyon, F-69007, Lyon, France.

^5^Georg-Speyer-Haus, Institute for Tumor Biology and Experimental Therapy, Frankfurt, Germany. ^6^German Cancer Consortium (DKTK), partner site Frankfurt/Mainz, Frankfurt, Germany.

^7^German Cancer Research Center (DKFZ), Heidelberg, Germany.

^8^Research Group for Gene Modification in Stem Cells; Division of Veterinary Medicine, Paul-Ehrlich-Institut, Langen, Germany

^9^Université Côte d’Azur, INSERM, C3M, Nice, France.

^10^German Cancer Consortium (DKTK), partner site Heidelberg, Heidelberg, Germany.

*joint first authors

#corresponding author: Christian Buchholz, E-mail: [christian.buchholz@pei.de](mailto:christian.buchholz@pei.de);

**Content**

Fig. S1: Gating strategies and fractions of human lymphocytes in PBMC-transplanted mice (page 3)

Fig. S2: Exemplary FACS plots of CAR^+^ T cells (page 4)

Fig. S3: Detection of Raji and B cells in different organs (page 5)

Fig. S4: Gating strategy and engraftment of human cells in CD34+ humanized mice (page 6)

Fig. S5: Immunohistochemistry to detect CD4^+^ lymphocytes (page 7)

**
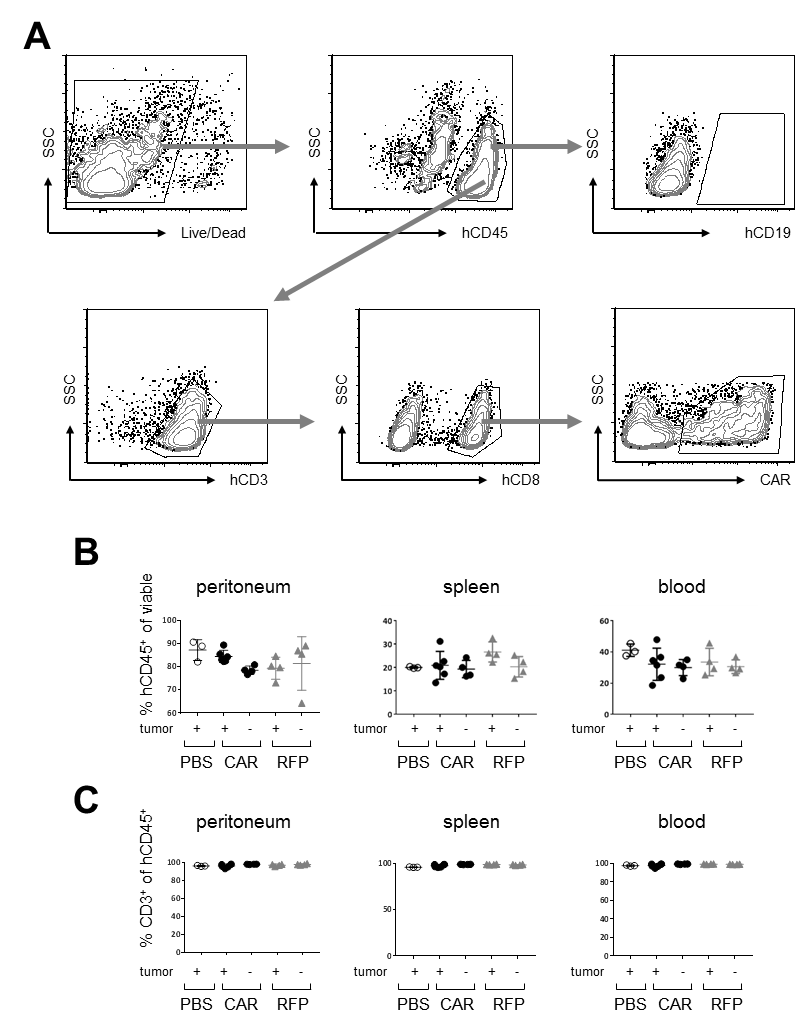
**

**Fig. S1: Gating strategies and fractions of human lymphocytes in PBMC-transplanted mice**

(**A**) Gating strategy. Living cells were hierarchically gated for human CD45, CD3, CD8 and CAR expression. Percentage of CD19^+^ cells was determined on CD45^+^ cells. (**B-C**) Cells isolated from the peritoneal cavity (peritoneum), spleen, or blood were evaluated by flow cytometry for the percentages of human CD45^+^ among all viable single cells (**B**) and of human CD3^+^ cells (**C**) within the fraction of human CD45^+^ cells. Data represent mean ± SD for all groups (CD8-LV^CD19CAR^ + tumor: n=6, PBS: n=3; all other: n=4).


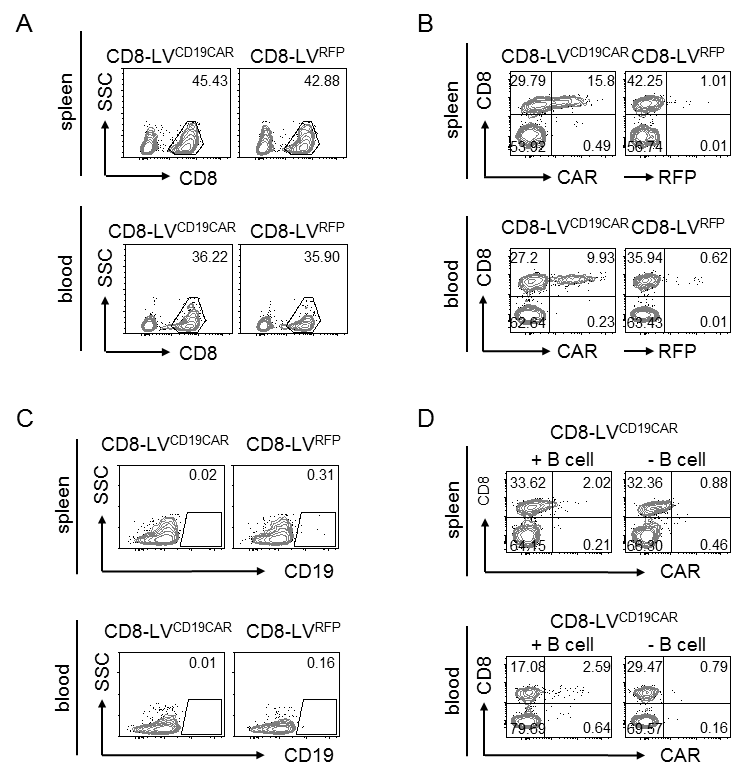


**Fig. S2: Exemplary FACS plots of CAR^+^ T cells**

Exemplary flow cytometry density plots of the data shown in Fig 1D-G for cells isolated from blood and spleen. (**A**) CD8 expression was analyzed within CD3^+^ cells, (**B**) CD19CAR or RFP expression in CD8^+^ and CD8^-^ cells (**C**) CD19 expression within CD45^+^ cells and (**D**) CD19-CAR expression in CD8^+^ and CD8^-^ cells.

**Fig. S3: Detection of Raji and B cells in different organs**

Raji and B cells were distinguished by their CD45 levels. Selected flow density blots showing human CD19 against CD45 of peritoneum, spleen and blood of PBMC engrafted mice with or without Raji cells and injected with the vectors as described in Fig. 1. Data are representative including one mouse from the CD8-LV^RFP^-injected group showing detectable levels of Raji cells in the peritoneal cavity. Gating for CD45^high^/CD19^+^ B-cells (blue) from PBMC and CD45^low^/CD19^+^ Raji tumor cells (red). Percentages shown are related to all cells.

**
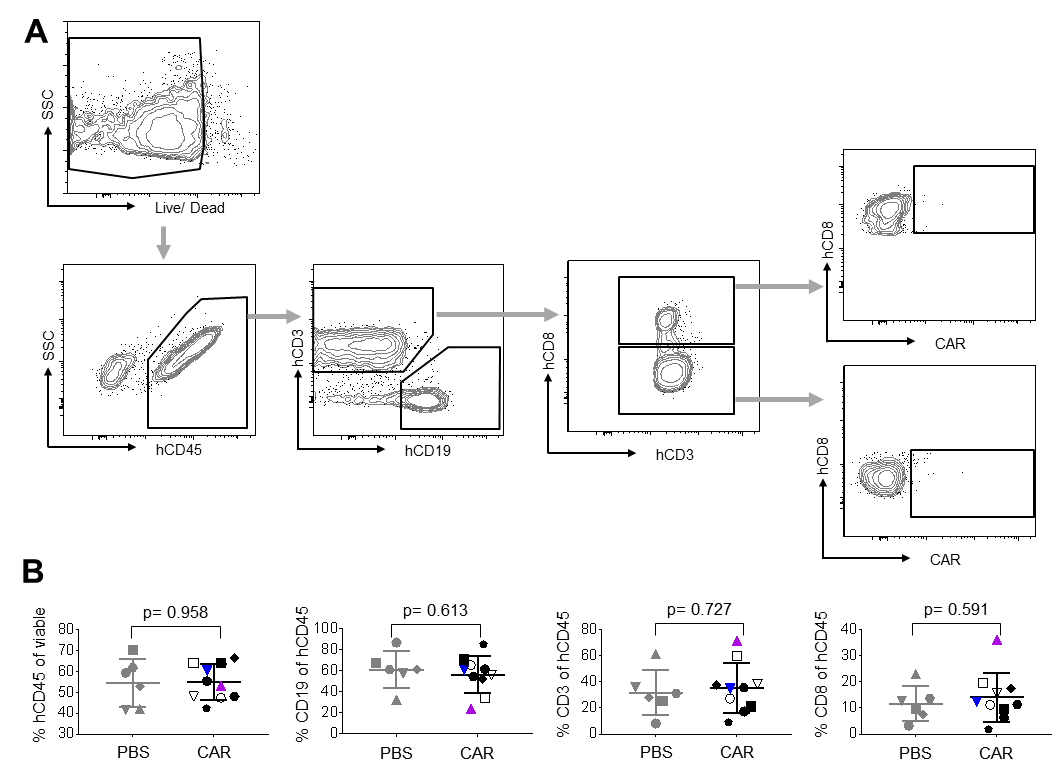
**

**Fig. S4: Gating strategy and engraftment of human cells in CD34+ humanized mice**

Additional information to Fig 2. **(A**) Gating strategy. Spleen, blood and bone marrow samples were used for FACS analysis after red blood cell lysis. Gating is indicated by the grey arrows starting with living cells which were hierarchically gated for human CD45, CD19, CD3, CD8 and CAR expression. CAR Expression was determined on CD8^+^ and CD8^-^ cells, respectively. (**B**) Initial human engraftment levels of CD45+, CD19+, CD3+ and CD8+ determined in peripheral blood samples of PBS and CD8-LV^CD19CAR^-injected mice before experimental start. Statistical calculations confirm an unbiased distribution of the individual mice to the two groups. All icons according to Fig. 2.

**
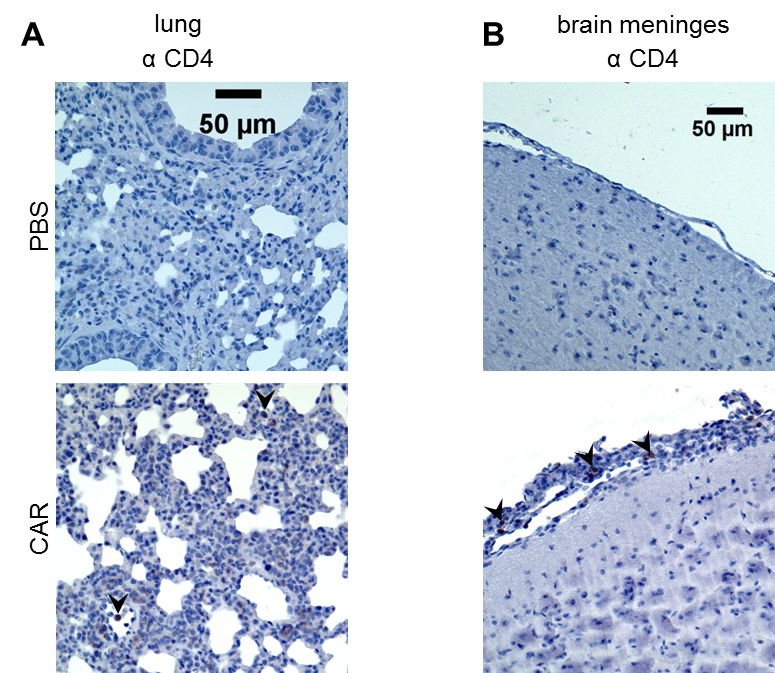
**

**Fig. S5: Immunohistochemistry to detect CD4^+^ lymphocytes**

Immunohistochemistry of paraffin embedded sections from the lung (A) and brain meninges (B) of the PBS control mouse M32 and the CAR^+^ mouse M16 stained with antibody clone 4B12 to detect CD4 (αCD4).
