# Supplementary material for: In vivo generation of human CD19‐CAR T cells results in B‐cell depletion and signs of cytokine release syndrome
Source: EMBO Mol Med. 2018 Sep 17;10(11):e9158. doi: 10.15252/emmm.201809158 (PMC6220327; doi:10.15252/emmm.201809158)
Supplement: Supplementary file 2 — Expanded View Figures PDF [file EMMM-10-e9158-s002.pdf]

Expanded View Figures

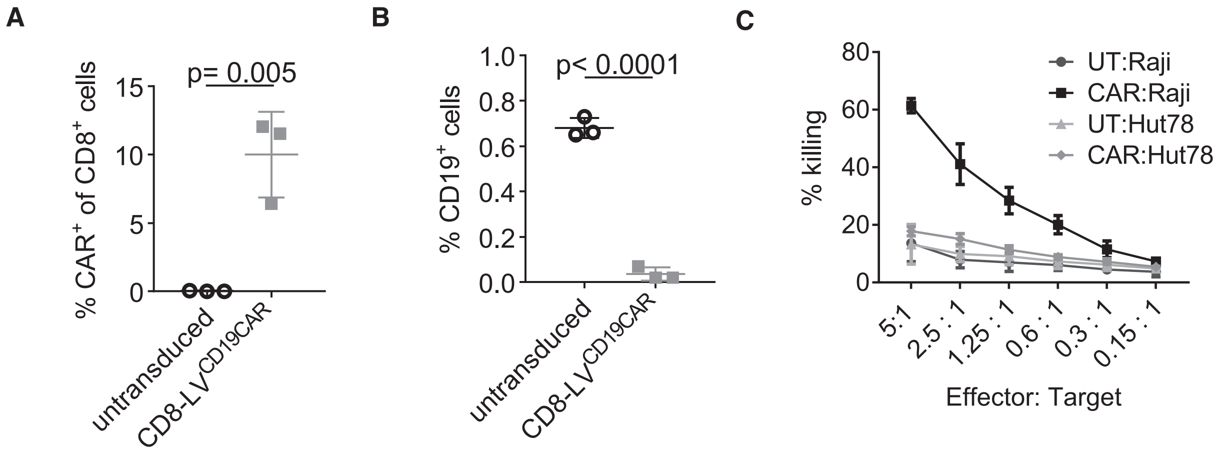

Figure EV1. ***Ex vivo* CAR T-cell generation.**

A Quantitative data of Fig 1A showing the percentages of CAR$^+$ cells of three different donors. Mean values ± SD are shown with n = 3. Statistical evaluation of the data was performed using two-tailed unpaired t-test.

B CAR T cells eliminate CD19$^+$ B cells. PBMC were activated for 3 days and incubated with CD8-LV^{CD19CAR}. Expression of CD3 and CD19 was then analyzed by flow cytometry. The percentage of CD19$^+$ B cells for n = 3 with mean ± SD is shown. Statistical significance was determined by two-tailed unpaired t-test.

C Selective killing of CD19$^+$ Raji tumor cells by *ex vivo*-generated CAR T cells (CAR) or untransduced T cells (UT) with CD19$^+$ Raji cells, or as control CD19$^-$ Hut78 cells. Mean values ± SD are shown with n = 3.

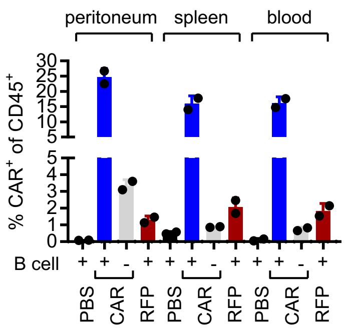

Figure EV2. Detection of CAR T cells in PBMC-transplanted mice.
Summary of CAR T-cell detection in PBMC-transplanted NSG by flow cytometry for the same two individual mice of each group shown in Fig 1C. Percentage of CAR$^+$ cells of CD45$^+$ cells is shown.

© 2018 The Authors

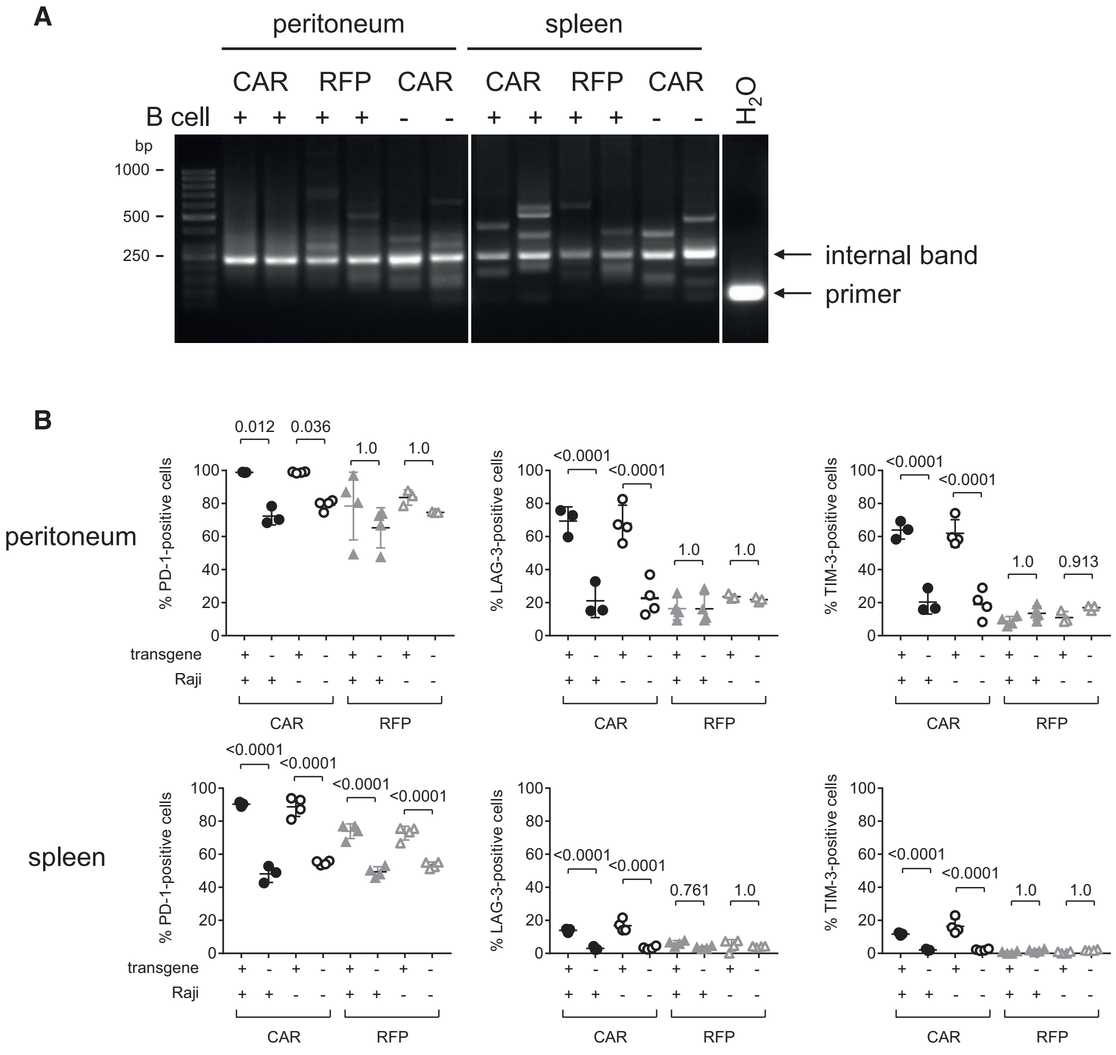

Figure EV3. Clonality and exhaustion of *in vivo*-generated CAR T cells.

A Clonality analysis by amplifying vector sequences integrated in genomic DNA. LM-PCR detecting the integrated vector in genomic DNA purified from peritoneal and spleen cells harvested from PBMC-transplanted mice injected with CD8-LV^{CD19CAR} (CAR) in the presence or absence of B cells or CD8-LV^{RFP} (RFP). Two mice of each group were analyzed. The internal control band is indicated as well as primer dimers in the water control (H_2O).

B Cells isolated from peritoneal cavity or spleen of human PBMC-transplanted NSG mice (± i.p. transplanted Raji cells) treated with CD8-LV^{CD19CAR} (CAR) or CD8-LV^{RFP} (RFP) were analyzed for expression of exhaustion markers by flow cytometry. CD8$^+$ cells from viable human CD3$^+$ cells were gated for transgene-positive (CAR$^+$ or RFP$^+$) and transgene-negative (CAR$^-$ or RFP$^-$) cells. These two cell populations were then separately gated for expression of PD-1, LAG-3, and TIM-3. For the four experimental groups, percentages of positive cells for each exhaustion marker are shown for transgene-positive and transgene-negative CD8$^+$ cells. Mean values ± SD are shown. N = 3 in samples with closed circles, n = 4 in samples with open circles or closed triangles, while for samples with open triangles, n = 3 for peritoneum and n = 4 for spleen. Statistical evaluation of the data was performed using one-way ANOVA test with Bonferroni correction.

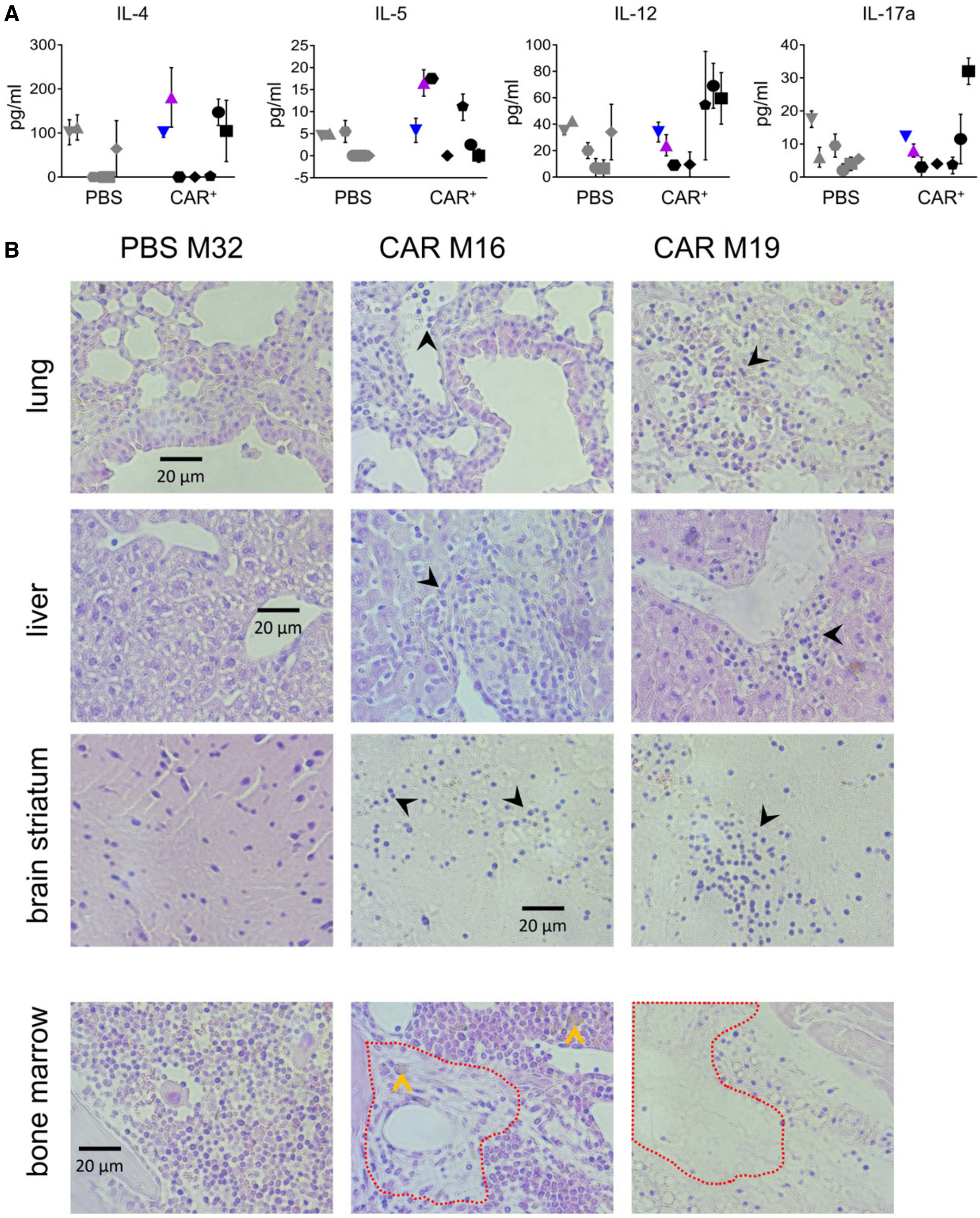

Figure EV4. Cytokines and organ infiltration.

A Levels of further cytokines in plasma of individual mice obtained 7 weeks after vector injection. The distinct symbols used for each individual mouse are identical to the ones used in Fig 2. Mean values of n = 2 technical replicas.

B Hematoxylin/eosin staining of paraffin-embedded sections from lung, liver, brain striatum, and bone marrow, of the PBS-injected control mouse M32 or the CD8-$^{LVCD19\text{-}CAR}$-injected M16 and M19 animals. Areas of hematopoietic cell depletion in bone marrow are indicated by the red-dashed line and acellular debris by the yellow arrows. Infiltrating lymphocytes in the other tissues are labeled by black arrows.

 © 2018 The Authors

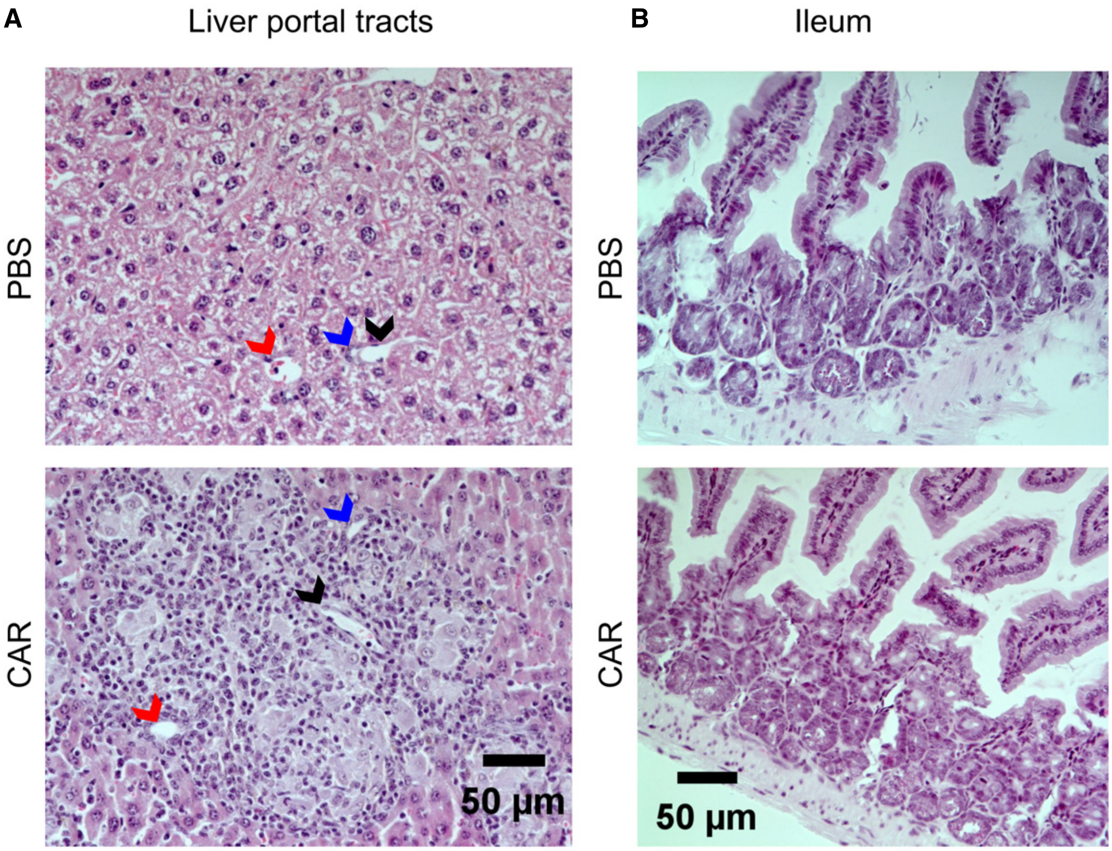

Figure EV5. Analysis for signs of GvHD.

A, B Hematoxylin/eosin staining of paraffin-embedded sections from liver portal tracts (A) and ileum (B) of the PBS control mouse M32 and the CAR$^+$ mouse M16. Arrows point to bile ducts (blue), arterial sinus (red), and portal vein (black).

© 2018 The Authors
